# Novel High-Performance Functionalized and Grafted Bio-Based Chitosan Adsorbents for the Efficient and Selective Removal of Toxic Heavy Metals from Contaminated Water

**DOI:** 10.3390/polym16121718

**Published:** 2024-06-16

**Authors:** Mohammad Monir, Rasha E. Elsayed, Rasha A. Azzam, Tarek M. Madkour

**Affiliations:** 1 Department of Chemistry, School of Sciences and Engineering, The American University in Cairo, AUC Avenue, New Cairo, Cairo 11835, Egypt; mohammadmonir@aucegypt.edu (M.M.); r.essam@aucegypt.edu (R.E.E.); 2 Department of Chemistry, Faculty of Science, Helwan University, Cairo 11795, Egypt; rasha_azzam@science.helwan.edu.eg

**Keywords:** chitosan, heavy metals, functionalized polymers, grafting copolymerization, adsorption

## Abstract

Novel functionalized and/or grafted crosslinked chitosan adsorbents were synthesized and used to remove several toxic heavy metal ions such as nickel, lead, chromium, and cadmium ions from contaminated water. The chitosan biopolymer was functionalized by maleic anhydride (CS_MA) acting also as a crosslinking agent. Glutaraldehyde-crosslinked chitosan (CS_GA) grafted with poly(methyl methacrylate) (CS_MMA) was also synthesized. The synthesized adsorbents were characterized using a variety of analytical techniques such as SEM, TGA, and FTIR, which confirmed their chemical structures and morphology. The adsorption capacity of the adsorbents was analyzed under various conditions of contact time, adsorbent dose, initial concertation, temperature, and pH and evaluated against those of pure chitosan (CS) and the crosslinked chitosan(CS_GA). The ultimate removal conditions were 0.5 g/100 mL adsorbent dose, an initial metal ion concentration of 50 ppm, a temperature of 45 °C, and pH 9. CS_MMA had the highest removal percentages for all metal ions, ranging from 92% to 94%. The adsorption was demonstrated to fit a pseudo-first-order model that followed a Langmuir adsorption isotherm. The results highlight the capacity of the synthesized polymers to efficiently remove major toxic contaminants at low cost from contaminated water, present especially in low-income areas, without harming the environment.

## 1. Introduction

The extensive use of different heavy metals in industrial applications and the discharge of industrial wastewater into water streams have resulted in major devastation of the health and environment of many communities, especially low-income ones [1,2]. Although heavy metals are commonly found in trace amounts in wastewater streams, such traces are still hazardous to the ecosystems [3,4]. The World Health Organization (WHO) listed eleven toxic heavy metal ions in wastewater effluents that are extremely harmful to human and animal health. Among these elements are nickel, lead, chromium, and cadmium, with maximum acceptable limits of 0.02, 0.01, 0.05, and 0.003 mg/L, respectively, in wastewater [5]. The toxicity level of these metals for the environment follows the order of Ni < Cr < Pb < Cd [5,6]. The toxic effects of these metals on human health range from gastrointestinal and kidney dysfunction to immune system dysfunction, and possibly cancer in some cases [7]. Accordingly, it is necessary to purify the discharged contaminated water before its discharge into the environment.

Over several decades, diverse traditional chemical and physical techniques have been developed for the removal of such heavy metals from wastewater [8,9,10,11]. Unfortunately, these traditional techniques have some drawbacks, such as low removal efficiency as well as high operational costs [12]. Consequently, new treatment methodologies have been developed for more efficient uptake of heavy metals, such as reverse osmosis [13], photocatalysis [14], and membrane separation [15]. Among these various treatment processes, adsorption presents the most common technique for removing heavy metals due to its efficiency in removing heavy metal ions at low concentrations, its operational flexibility, and its low cost [12,16].

Polymeric adsorbents have been among the important and prime choices for wastewater treatment applications due to their facile availability, cost-effectiveness, biocompatibility, and biodegradability [17]. Modified chitosan structures through crosslinking, grafting, and blending have been reported. For example, Vijaya et al. [18] investigated the efficacy of chitosan-coated silica (CCS) and chitosan-coated calcium alginate (CCCA) adsorbents on the removal of nickel ions from wastewater. On the other hand, successful attempts based on chitosan-based polymeric adsorbents were reported for the removal of lead ions from aqueous solutions [19,20]. Recently, Khanniri et al. [20] evaluated the potential of chitosan/bacterial yeast blends as biosorbents for lead removal. A novel pyridinium-functionalized magnetic chitosan adsorbent was synthesized through the amination of magnetic chitosan with diethylenetriamine, followed by quaternization of the product with pyridine for the magnetic separation of toxic hexavalent chromium in aqueous solutions [21]. An efficient removal of Cr(VI) was observed with a maximum capacity of 176 mg/g at a quite acidic pH value of 3. Chitosan/sulfhydryl-functionalized graphene oxide composites were found to be an effective adsorbent for Cd^+2^ ions uptake from aqueous solutions [22].

Modification of the chitosan surface via grafting copolymerization has also gained great attention lately for the effective removal of heavy metals and other contaminants from contaminated water. Shankar et al. [23] attempted to graft-copolymerize glutaraldehyde-crosslinked chitosan with acrylonitrile using cerium ammonium nitrate initiator for chromium (VI) adsorption. Bal et al. [24] employed crotonic acid as an alternative monomer in the grafting copolymerization of chitosan for the efficient removal of Cu(II) ions from water.

The aim of this research is to develop tailored-design chitosan-based polymeric adsorbents with low-cost, facile application techniques and superior adsorption efficiency of a host of toxic heavy metal ions from contaminated water.

## 2. Materials and Methods

### 2.1. Materials

Chitosan, with an average molecular weight (Mn) of 250 kDa and a deacetylation degree of 85%, was obtained from natural resources and purchased from Alpha Chemicals, Cairo, Egypt. Glutaraldehyde (GA), maleic anhydride (MA), and methyl methacrylate (MMA) were all purchased from El Nasr Pharmaceutical Chemicals, Cairo, Egypt. Ammonium persulfate and sodium hydroxide of analytical chemical grade were purchased from Delta Chemical Company, Cairo, Egypt. Acetic acid was purchased from Sigma Aldrich, Aachen, Germany. Ethanol was obtained from Carlo Erba, Paris, France. Ni(II), Pb(II), Cd(II), and Cr(VI) chlorides were brought from Loba Chemie, Delhi, India.

### 2.2. Methodology

#### 2.2.1. Synthesis of Maleic Anhydride-Functionalized Chitosan (CS_MA)

**Figure 1:**
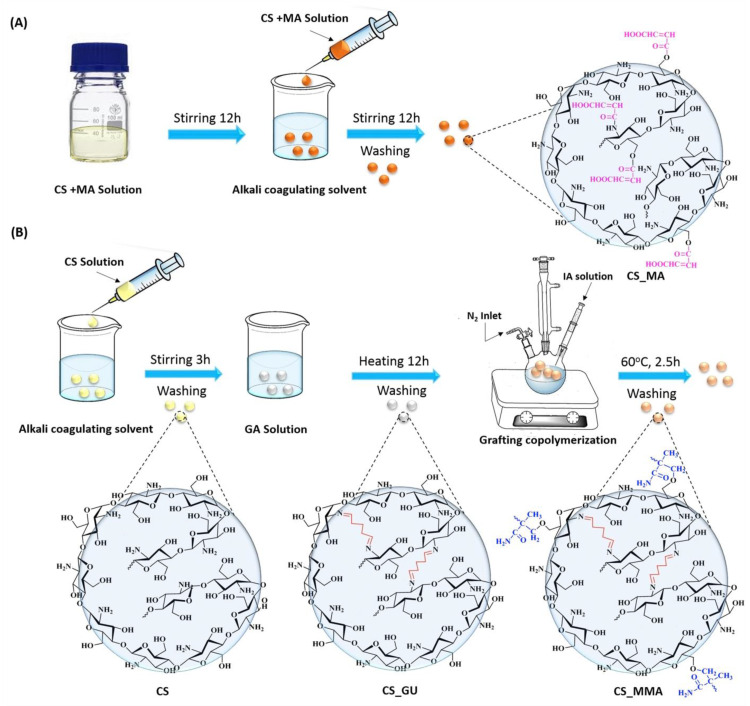
Schematic representation of the synthesis of (**A**) chitosan beads crosslinked and functionalized with maleic anhydride (CS_MA), and (**B**) chitosan beads crosslinked with glutaraldehyde (CS_GA), and graft copolymerized with poly(methyl methacrylate) (CS_MMA).

**Figure 2:**
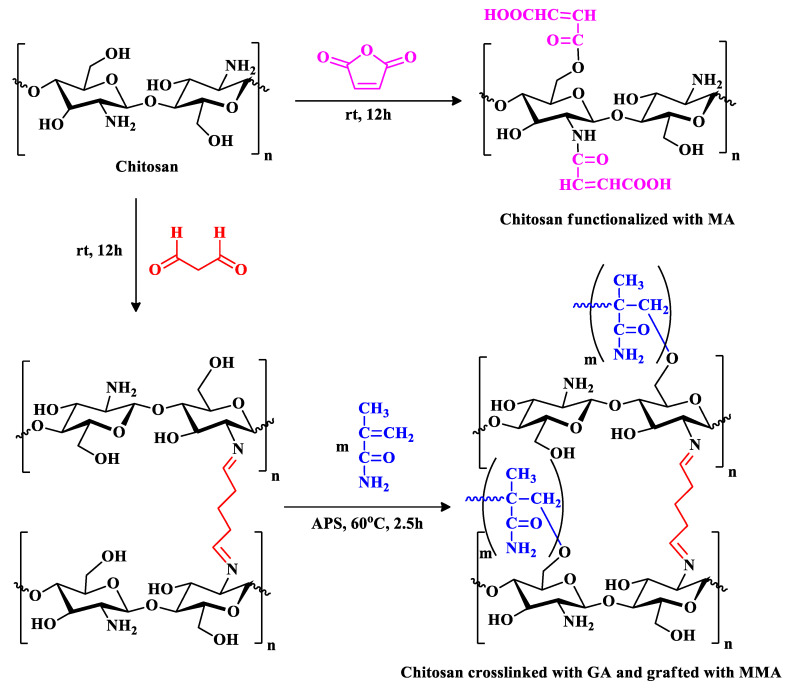
Synthetic pathway of MA-functionalized chitosan (CS_MA) and MMA-grafted chitosan (CS_MMA).

To synthesize maleic anhydride-functionalized chitosan, 3 g of chitosan were dissolved in 100 mL aqueous acetic acid solution (1% *v*/*v*) and allowed to stir for 24 h. Parallel to that, 3 g of maleic anhydride was also allowed to dissolve in 100 mL of aqueous acetone solution (25% *v*/*v*). The maleic anhydride solution was then poured into the chitosan solution and allowed to stir for 24 h, Figure 1A. A 20-gauge syringe was used to formulate the polymeric beads in 1 L of 0.5 M sodium hydroxide solution. The chemical structure of the resultant polymeric sample (CS_MA) is shown in Figure 2. The beads were left for 24 h in the sodium hydroxide solution and were then washed multiple times using distilled water to remove any unreacted materials.

#### 2.2.2. Chitosan Beads Crosslinked with Glutaraldehyde and Grafted with Methyl Methacrylate (CS_MMA)

Crosslinked chitosan beads were prepped by dissolving 3 g of chitosan in 100 mL aqueous acetic acid (1% *v*/*v*) and stirred constantly for 24 h until a homogeneous viscous solution was observed. The solution was then added dropwise into 1 M NaOH solution using a 20-gauge syringe needle and left overnight to neutralize the acid. The chitosan beads at this point were washed many times with distilled water to remove any unreacted materials and/or sodium hydroxide residues from the sample. The cross-linking process was performed using glutaraldehyde as the crosslinking agent by placing the wet beads in 2.5% glutaraldehyde with a ratio of 1:1.5 by weight. The reaction was then left to stir for 24 h, and the beads were filtered and washed again with distilled water to dispose of any unreacted glutaraldehyde. The glutaraldehyde-crosslinked chitosan was produced, as demonstrated in Figure 1B. The chemical structure of the resultant polymeric sample (CS_GA) is shown in Figure 2.

After the crosslinked chitosan was dried, 1 g of the beads was transferred along with 0.6 g of ammonium persulfate to a 250 mL three-necked flask equipped with a magnetic stirrer, gas inlet system, a reflux condenser, and nitrogen atmosphere. The mixture was stirred for one hour at 60 °C to facilitate radical formation of the chitosan chains (Figure 1). Three grams of methyl methacrylate was slowly added into the mixture with continuous stirring and a flow of oxygen-free nitrogen gas for two and a half hours (Figure 2). After the graft copolymerization was completed, the grafted beads, CS_MMA, were washed several times with distilled water to remove any unreacted reagents. The grafting of chitosan was evaluated based on the grafting yield (*Y*%) and grafting percentage (*G*%) and calculated as follows [25]:

(1) $$G\left(\%\right)=\frac{w_{2}-w_{1}}{w_{1}}\;\times \;100$$

(2) $$Y\left(\%\right)=\frac{w_{2}-w_{1}}{w_{3}}$$

where *W*_1_, *W*_2_, and *W*_3_ indicate the mass of crosslinked chitosan, grafted copolymer, and methyl methacrylate, respectively. In this research, the *G%* and *Y%* obtained for the grafted sample were 215% and 73%, respectively.

#### 2.2.3. Characterization of the Modified Chitosan Beads

Characterization studies were conducted on the four prepared chitosan samples (CS, CS_MA, CS_GA, and CS_MMA). Morphological investigation was performed using SEM SUPRA 55 LEO SEM instrumentation by Carl Zeiss, Jena, Germany. Gold sputtering HUMMER 8.0 by Anatech LTD, Sparks, NV, USA, was used to cover the sample’s surfaces with cold to improve the images’ quality by making the sample more conductive. Gold sputtering was applied at 15 mA for one minute. To determine the thermal degradation behavior of the chitosan samples, thermogravimetric analysis was performed using Perkin Elmer TGA 7 Thermogravimetric Analyzer by PerkinElmer, Inc., Waltham, MA, USA. The mass of the sample was in the range of 10–12 mg. The sample pan was placed inside the balance system equipment, and the temperature was elevated from 25 to 750 °C at a heating rate of 5 °C per minute. The chemical composition of the chitosan samples was also studied using Fourier-transform infrared spectroscopy Thermo Scientific- NICOLET 380 FTIR spectrometer by Nicolet, Thermo Fisher Scientific, Waltham, MA, USA. The experiment was carried out at room temperature and utilized KBr pellets in the range of 4000–400 cm^−1^ with a spectral resolution of 4 cm^−1^.

#### 2.2.4. Removal of Ni(II), Pb(II), Cd(II), and Cr(VI) Ions

The uptake of Ni^2+^, Pb^2+^, Cd^2+^, and Cr^6+^ ions in their individual aqueous solutions was studied under diverse conditions (initial concentration of the heavy metals, adsorbent dose, and temperature). Batch adsorption experiments were performed at pH 9, which represented the best pH that yielded maximum uptake for each heavy metal in the screening study. A pre-weighted sample of the modified chitosan beads was immersed in 100 mL heavy metal solution of a certain concentration and analyzed using high-pressure liquid chromatography (HPLC) for different time intervals. The equilibrium adsorption capacity for the metal ions was determined and calculated using the following equation:

(3) $$q_{e}=\frac{(C_{0}-C_{e})\;V}{m}$$

where *q_e_* is the equilibrium adsorption capacity (mg/g), *C_0_* is the initial metal ion concentration (ppm), and *C_e_* is the equilibrium heavy metal concentration (ppm). *V* is the volume of solution (mL), *m* is the molar mass of metal ion (g mol^−1^), and *m* is the mass of chitosan adsorbent (g) [26].

Removal efficiency (*%R*) of the modified chitosan beads was also calculated as follows:

(4) $$\%R=\frac{(C_{0}-C_{e})\;}{C_{0}}$$

In the initial concentration study, 0.5 g of the adsorbent was soaked in 100 mL of the various aqueous solutions of the different metal ions. The initial concentrations of 5, 20, 50, and 100 ppm were used to study the effect of the contaminant’s initial concentration on the percent removal. The study was carried out at pH 5.8 and a temperature of 25 °C. The influence of chitosan adsorbent dose on the adsorption and removal of the four metal ions was conducted with different adsorbent masses of 0.2, 0.5, 0.8, and 1 g in 100 mL of aqueous solutions of the metal ions. The initial concentration of the contaminants in this part of the study was chosen to be 50 ppm and at a temperature of 25 °C. The temperature effect on the adsorption of metal ions on the surface of the modified chitosan beads was carried out at different temperatures 25, 45, 65, and 85 °C.

#### 2.2.5. Equilibrium Studies

Adsorption isotherms were fitted using three different isotherm models, namely, linear, Freundlich, and Langmuir models, using the following equations, respectively.

(5) $$q_{e}=K_{e}C_{e}$$

(6) $$q_{e}={K_{F}C_{e}}^{1/n}$$

(7) $$q_{e}=\frac{q_{m}C_{e}K_{e}}{C_{e}K_{e}+1}$$

where *K_e_* is the equilibrium constant for the adsorption process, *C_e_* is the adsorbate concentration (ppm), *q_m_* is the maximum adsorption capacity, and *K_f_* and *n* present Freundlich constants. Different measures were taken at 25, 45, 65, and 85 °C to determine the thermodynamic properties, including enthalpy (∆H), entropy (∆S), and Gibbs free energy (∆G). The equilibrium constant was obtained from the following equation [26]:

(8) $$K_{d}=\frac{q_{e}}{C_{e}}$$

∆H, ∆S, and ∆G were calculated according to

∆G = −*RT* ln *K_d_* (9)

(10) $$\ln K_{d}=\frac{\Delta S}{R}-\frac{\Delta H}{RT}$$

where *T* is the temperature in Kelvin, and *R* is the ideal gas constant. Based on the final equation, the plot of *K_d_* versus 1/*T* should produce a straight line that has a slope of (∆H/*RT*) and intercept of (∆S/*R*).

#### 2.2.6. Kinetic Modeling

Pseudo-first-order and pseudo-second-order kinetics models were investigated to predict the kinetic uptake profiles of the different metal ions according to [27,28]:

(11) $$\log(q_{e}-q)=\log\left(q_{e}\right)-\frac{K_{1}}{2.303}t$$

(12) $$\frac{t}{q_{e}}=\frac{1}{{K_{2}q_{e}}^{2}}+\frac{1}{q_{e}}t$$

where *K*_1_ and *K*_2_ are the respective rate constants of the pseudo-first-order and pseudo-second-order models, and *q* is the uptake capacity at time t.

To explore the applicability of the mechanism for intra-particle diffusion, the Weber and Morris intra-particle diffusion model was examined [29].

(13) $$q=K_{id}t^{0.5}+c$$

where *K_id_* is the constant of intra-particle diffusion, and *c* is a constant reflecting the boundary layer effect.

#### 2.2.7. Validating the Effect of pH

As described earlier, the metal ion removal investigation was carried out at pH 9, which was determined to be the most suitable medium for the adsorption of the metal ions on the surface of the chitosan beads. To ensure that the alkaline medium at pH 9 had no impact on the removal of the metal ions from the aqueous solutions through the precipitation of the ions, four solutions of the different metal ions were prepared and adjusted for pH 9. The samples were then left for 24 h at 25 °C. The heavy metal ion concentrations were measured and found to be constant. This indicated that the pH did not cause any precipitation of the ions and, accordingly, it had no impact on the adsorption study.

#### 2.2.8. Statistical Analysis

All experiments were conducted three times at a minimum, with the obtained data presented as mean standard deviation (SD). Statistical analysis was performed using a one-way ANOVA test, followed by Tukey’s multiple comparison test for all data obtained from the examination of the modified chitosan. All tests were calculated using GraphPad Prism Software Version 6 and presented as * *p* < 0.05, ** *p* < 0.01, *** *p*< 0.001, and **** *p*< 0.0001.

## 3. Results and Discussion

### 3.1. Chemical and Physical Characterization

#### 3.1.1. SEM Imaging

**Figure 3:**
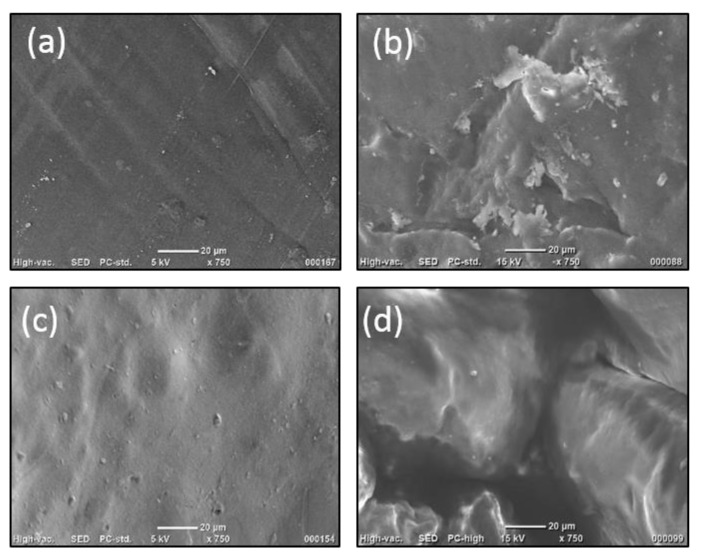
SEM Imaging of (**a**) CS, (**b**) CS_GA, (**c**) CS_MA, and (**d**) CS_MMA.

Figure 3 demonstrates the SEM images of CS, CS_GU, CS_MA, and CS_MMA, respectively. For all the samples, images were taken prior to any adsorption experimentations at a magnification of 750. As is obvious from 3a, the sample exhibits smooth texture with no apparent pore formation. CS_GU demonstrated some random wavy points on the surface, while SEM of CS_MA showed a smoother morphology. SEM micrographs clarified the influence of grafting copolymerization of the prepared chitosan adsorbent CS_GU with methyl methacrylate. Interestingly, it was observed that the surface of the adsorbent became non-homogeneous with some cavities. This is probably due to hydrophilic interactions between water molecules with the grafted side chains [30]. After drying, the water molecules left such cavities on the surface of the dried beads.

#### 3.1.2. TGA

**Figure 4:**
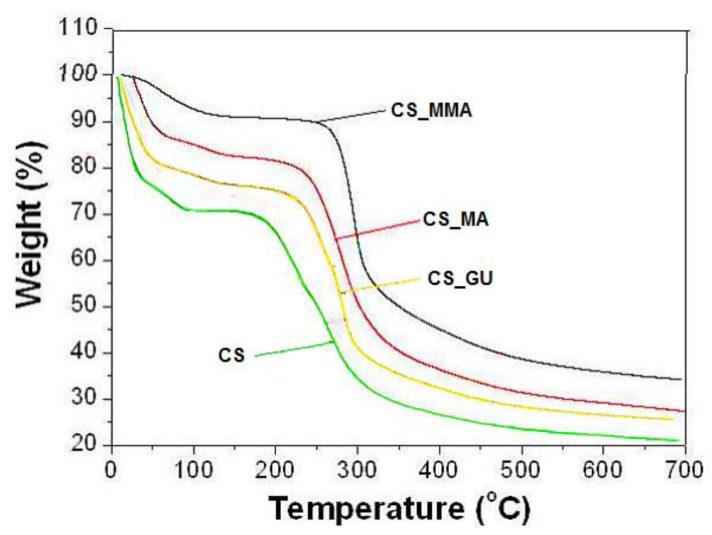
TGA of CS, CS_GU, CS_MA, and CS_MAA samples.

TGA analysis of the four synthesized samples was performed, and the results are shown in Figure 4. This figure indicates the presence of two stages of weight loss for all samples. The first one shows a weight loss of approximately 10% in the range of 45–100 °C, which corresponds to moisture loss [31], while the second one takes place in the range of 200 to 300 °C, due to chitosan deacetylation and the elimination of volatile products with a weight loss of 35% [32]. A slight change in the decomposition temperature took place with the crosslinking of the chitosan sample as well as the grafting copolymerization of the sample, due to the increased thermal stability resulting from the formation of crosslinks between the chains. Interestingly, the type of crosslinking had no bearing on the change in the decomposition temperature since both CS_GU and CS_MA had very similar decomposition temperatures and profiles. Finally, a complete breakdown of the main backbone of chitosan has started at around 300 °C. It can be concluded from the figure that the CS_MMA sample has the highest thermal stability among all other samples and that all samples are particularly stable at room temperatures. This is important, since these highly biodegradable samples need to be stable during the treatment of contaminated water, while they may be easily degradable when discarded via biodegradation processes.

#### 3.1.3. FTIR

**Figure 5:**
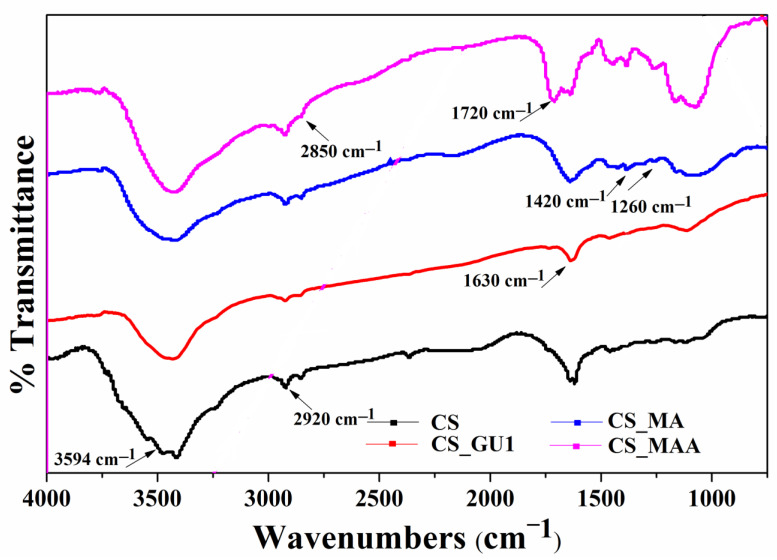
FTIR spectrums (a) of CS, CS_GU, CS_MA, and CS_MAA samples.

As shown in Figure 5, the FTIR spectra of the chitosan samples showed several peaks. To elaborate, the peak at 570 cm^−1^ is assigned to the out-of-plane NH and C-O bonds, while the band at 1180 cm^−1^ is assigned to C-O-C stretching. Additionally, two noticeable peaks located at 2920 cm^−1^ and 3594 cm^−1^ were assigned to the CH_2_ and OH stretching, respectively [33]. The CS_GU spectra show a peak around 1630 cm^−1^, which represents the stretching vibrations of C=N moiety in the Schiff’s base resulting from the reaction of chitosan and glutaraldehyde [34]. The reaction between maleic anhydride and chitosan is confirmed by the peaks at 1600, 1260, and 1420 cm^−1^, representing a ring-opening reaction of the maleic anhydride unit [35]. Nevertheless, the absorption band at 1475 cm^−1^ represents the C=C alkenes [35]. The absorption band at 1720 cm^−1^ is due to C=O stretching vibration of the ester bonds formed during the reaction [36]. Finally, the peak around 2850 cm^−1^ confirms the grafting process of methyl methacrylate [36].

### 3.2. Contaminant Removal under Various Operating Conditions

**Figure 6:**
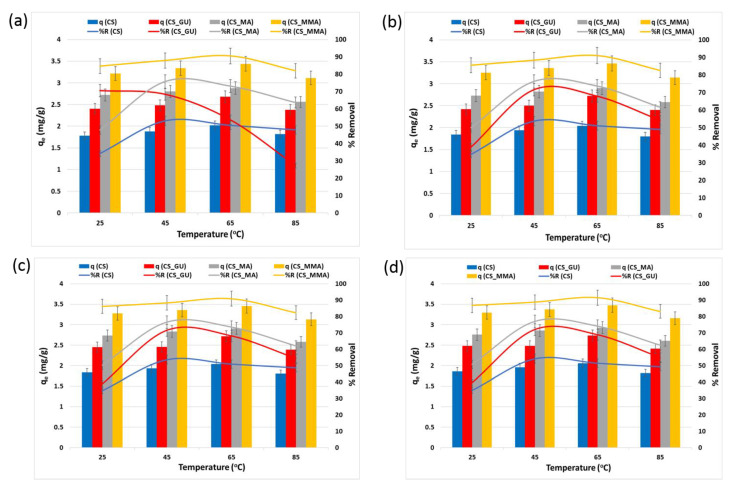
Equilibrium uptake capacity and percent removal of nickel (**a**), lead (**b**), chromium (**c**), and cadmium (**d**) at different temperatures. The experimental conditions were an ion concentration of 20 ppm and an adsorbent dose of 0.5 g/100 mL at pH 9.

**Figure 7:**
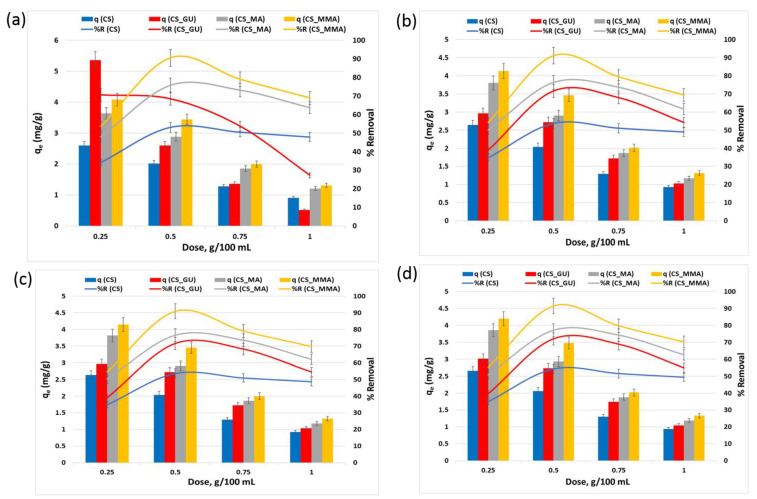
Equilibrium uptake capacity and percent removal of nickel (**a**), lead (**b**), chromium (**c**), and cadmium (**d**) at different adsorbent doses. The experimental conditions were an ion concentration of 20 ppm and a temperature of 45 °C at pH 9.

**Figure 8:**
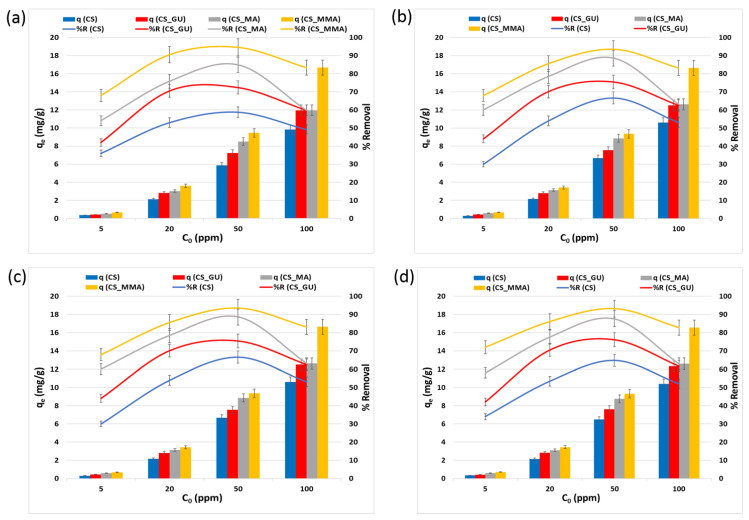
Equilibrium uptake capacity and percent removal of nickel (**a**), lead (**b**), chromium (**c**), and cadmium (**d**) at different initial concentrations. The experimental conditions were an adsorbent dose of 0.5 g/100 mL and a temperature of 45 °C at pH 9.

The synthesized chitosan samples were investigated for their efficiency in removing four heavy metal ions (nickel, lead, chromium, and cadmium) under various operating conditions, namely, the initial metal ion concentration, adsorbent dose, and temperature. Equilibrium uptake capacity, *q_e_*, and percent removal (*R%*) of the metal ions were determined using the kinetic uptake profiles. The kinetic profiles were studied under each condition to obtain *q_e_* and *R%*, as represented in Figure 6, Figure 7 and Figure 8.

#### 3.2.1. Impact of the Operating Temperature on *q_e_* and *R%*

The temperature influence on *q_e_* and *R%*, shown in Figure 6, is due to the kinetic energy activation of the metal ions, resulting in an acceleration of their mobilities, which enhances the ion adsorption on the surface of chitosan beads [37]. However, at higher temperatures, the metal ions become highly activated and easily collide with each other, resulting in a reduction in their ability to reach the adsorbent groups, which in turn diminishes both *q_e_* and *R%* [38]. It was noted that at 45 °C, all the samples achieved the greatest removal percentage, except for the CS_MMA, which had a minor increase in the removal percentage at 65 °C. The grafted chitosan sample (CS_MMA) showed a significant increment in the uptake when compared to the non-grafted sample (CS_GU) at 45 °C (** *p* < 0.01). Furthermore, the maleic anhydride-functionalized chitosan sample (CS_MA) also showed higher removal percentage as compared to the CS control sample (*** *p* < 0.001).

#### 3.2.2. Impact of the Adsorbent Dose on *q_e_* and *R%*

The increase in the adsorbent dose initially increased *R%* while reducing *q_e_*. However, further dose increases caused a reduction in both *q_e_* and *R%* due to the agglomeration effect of the adsorbent beads [37]. Basically, as more material is added, a lower number of the surface-active sites become accessible to the metal ions, see Figure 7. The adsorbent dose of 0.5 g/100 mL was found to be the optimum dose in this study for the removal of all tested heavy metals. Upon using the optimum dose, either chitosan grafting or crosslinking had a noteworthy impact on the removal of the tested heavy metals (** *p* < 0.01). Meanwhile, CS _MMA exhibited a noticeable increase in the uptake when compared to CS_MA and CS_GU (** *p* < 0.01), whereas no significant effect was observed on the metal on removal between CS_MA and CS_GU.

#### 3.2.3. Impact of the Initial Metal Ion Concentration on *q_e_* and *R%*

It can be noted from Figure 8 that the equilibrium uptake capacity of nickel, lead, chromium, and cadmium ions increased with the rise in the initial concentration, due to the increase in the driving force caused by the increase in the initial concentration [39,40]. Consequently, this should have led to a rise in *R%*. Nevertheless, the opposite was observed, as the increase in the initial concentration of the metal ions had an adverse impact on the percent removal. This could be explained on the basis that an enhanced adsorbent action results in attracting a greater number of the metal ions at higher initial ion concentrations, thus significantly covering the surface of the chitosan samples and resulting in a lower number of the surface-active sites able to adsorb the ions and remove them from the solution [41]. These results demonstrated that the initial ion concentration of 50 ppm corresponds to the optimum value of the percent removal. At such a concentration, functionalization of the chitosan adsorbents with maleic anhydride resulted in a significant increase in the uptake of the selected heavy metal when compared to the chitosan control (*** *p* < 0.001). Interestingly, the grafting of glutaraldehyde-crosslinked chitosan beads with methyl methacrylate noticeably affected on the removal of all tested heavy metals (*** *p* < 0.001). Therefore, CS_MMA samples exhibited the highest removal among all other chitosan samples, while no significant difference was observed between CS_MA and CS_MMA.

#### 3.2.4. Ultimate Adsorption Condition and Statistical Analysis

**Figure 9:**
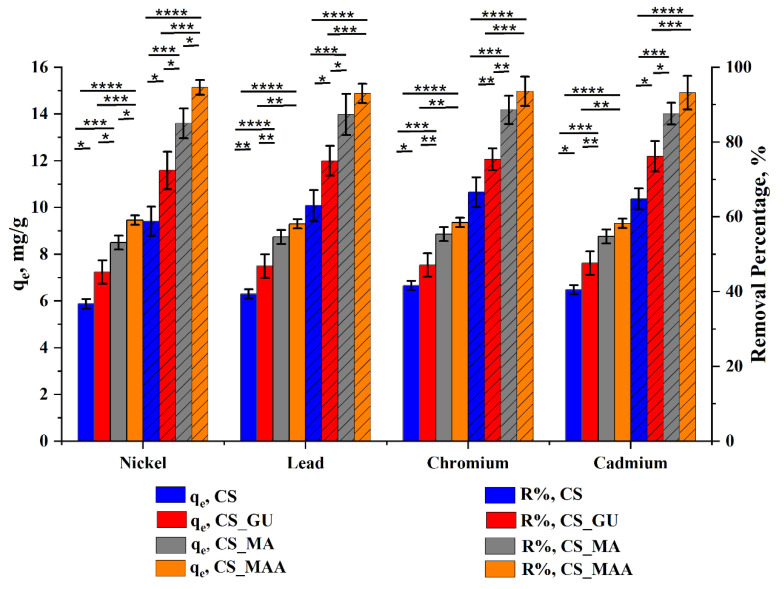
Optimum condition for maximum uptake of nickel, lead, chromium, and cadmium at an initial ion concentration of 50 ppm, an adsorbent dose of 0.5 g/100 mL, a temperature of 45 °C, and pH 9 (d). Data represent the mean ± SD. *p* values are for one-way ANOVA, followed by a Tukey’s multiple comparisons test (* *p* < 0.05, ** *p* < 0.01, *** *p* < 0.001, **** *p* < 0.0001).

Since the efficient removal of the various heavy metals in a cost-effective manner is the main purpose of this study, the best conditions for maximizing the uptake of these ions from contaminated water were determined to be an initial ion concentration of 50 ppm and an adsorbent dose of 0.5 g/100 mL, with a temperature of 45 °C (Figure 9).

The results of statistical data in Figure 7 demonstrated considerable significant differences in the removal percentage between the chitosan control sample and the rest of the prepared samples (up to **** *p*< 0.0001). After the chitosan crosslinking with glutaraldehyde, a significant increase in *q_e_* and *R%* of the tested heavy metal ions was observed (* *p*< 0.05). Surprisingly, graft copolymerization of the glutaraldehyde-crosslinked chitosan has appreciably affected *q_e_* and *R%* out of all the tested heavy metal ions (**** *p*< 0.0001). Similarly, the functionalization of chitosan with MA had a considerable effect on both *q_e_* and *R%* (up to *** *p*< 0.001).

Meanwhile, a significant difference between CS_MA and CS_MMA on the *q_e_* and *R%* of the nickel ions was observed (* *p* < 0.05), while in the case of lead, chromium, and cadmium, no significant change was observed (* *p* > 0.05). Based on the resultant statistical data, among all synthesized samples, methyl methacrylate-grafted chitosan (CS_MMA) showed the best performance as an adsorbent of nickel, lead, chromium, and cadmium heavy metal ions. Under these conditions, further kinetic and equilibrium studies were conducted.

### 3.3. Thermodynamic Analysis of the Adsorption Process

To elucidate the nature of the adsorption mechanism, various thermodynamic parameters, including the change in enthalpy (∆H), entropy (∆S), and Gibbs free energy (∆G), were calculated. Using the van ’t Hoff plot, Figure S1, the thermodynamic parameters of CS_MA and CS_MAA samples for the adsorption of the four heavy metals are demonstrated in Table 1, and thermodynamic parameters of the rest of the prepared samples CS and CS_GU are tabulated in Table S1. For the adsorption of the employed heavy metals using the different chitosan-based polymeric adsorbents, the negative free energy (ΔG) and the negative enthalpy (ΔH) indicate spontaneous exothermic processes for the CS and CS_GU systems [42]. Additionally, the negative free energy (ΔG) and the positive enthalpy (ΔH) indicate spontaneous endothermic processes for CS_MA and CS_MMA systems (Table 2). The small magnitude of ΔH implies that the adsorption of the metal ions on the surface of the different chitosan-based polymeric adsorbents took place through weak physisorption [43]. Furthermore, the equilibrium constants for the adsorption of the metal ions were more than unity, which indicates spontaneity. By inspecting the adsorption isotherms depicted in Figure S2, it can be concluded that these isotherms follow Langmuir isotherm behavior where multiple-layer surface coverage must have occurred.

### 3.4. Kinetic Study of the Adsorption of the Metal Ions

**Figure 10:**
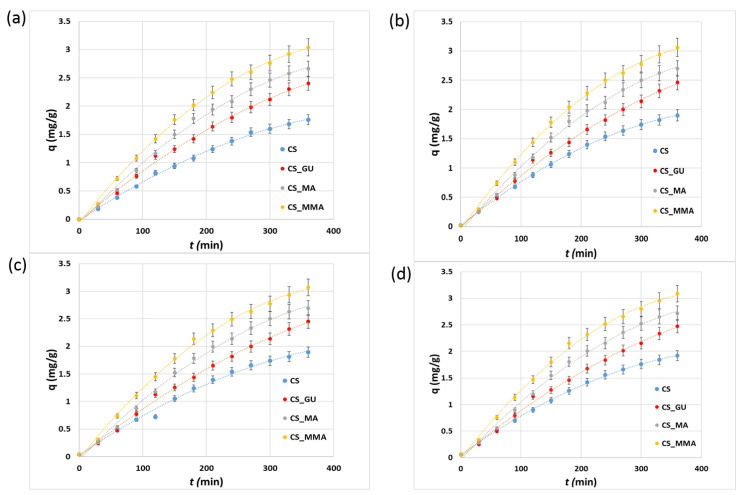
Kinetic profiles for the uptake of nickel (**a**), lead (**b**), chromium (**c**), and cadmium (**d**) by the different chitosan-based polymeric adsorbents.

The kinetic uptake profile for the adsorption of nickel, lead, chromium, and cadmium metal ions on the surface of the different chitosan adsorbents is shown in Figure 10. It can be observed that the adsorption capacity of CS_MMA towards the metal ions was higher than that of the other chitosan adsorbents after 360 min. Accordingly, the intermolecular forces between the ions and CS_MMA are expected to be high enough to increase their removal percentages. For estimating a kinetics model for the adsorption processes, all the profiles were fitted to the pseudo-first-order and pseudo-second-order models. The resultant linear plots are shown in Figures S3–S5, respectively. Such figures were utilized to forecast the kinetic parameters for the prepared CS_MA and CS_MAA shown in Table 2. The kinetic parameters of the other two samples, CS and CS_GU, are presented in Table S2. By scrutinizing the correlation factor (R^2^) for each model, it can be confirmed that the pseudo-first-order model best fits the adsorption of the metal ions, since it had higher R^2^ values for all systems, see Table 2. Using this model, the expected kinetic rate constants for the metal ion adsorption were very analogous in values showing equal adsorption rates. Since all the profiles were linear, the adsorption can be concluded to occur via intra-particle diffusion (Figure S5).

### 3.5. Influence of pH on Metal Ion Adsorption and the Adsorption Mechanism

**Figure 11:**
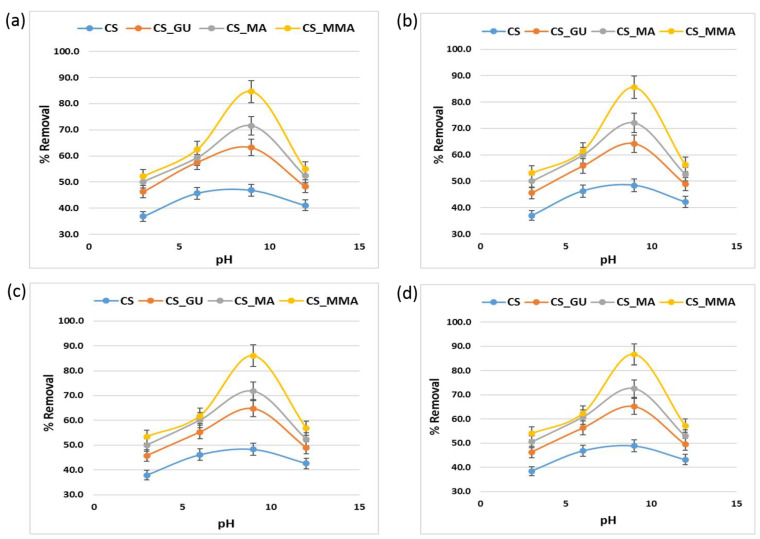
Influence of pH values on the percent removal of nickel (**a**), lead (**b**), chromium (**c**), and cadmium (**d**) using the different chitosan-based polymeric adsorbents.

**Figure 12:**
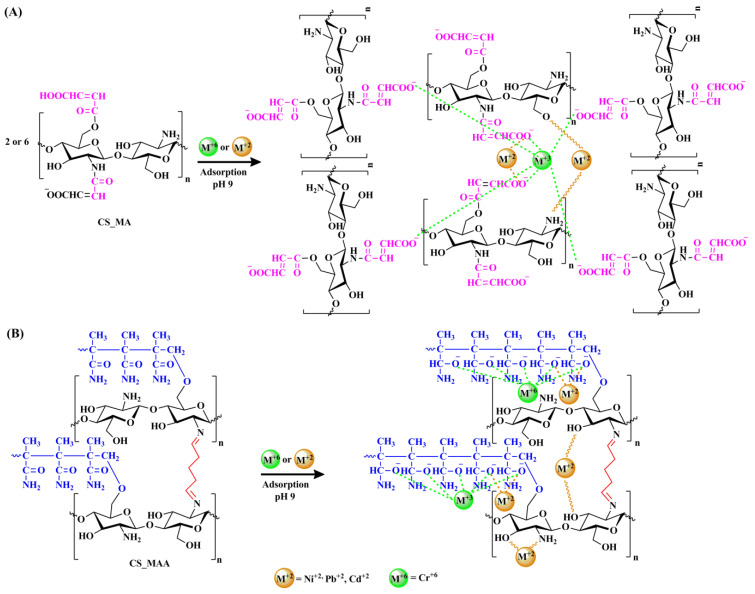
Possible interations between Ni^+2^, Pd^+2^, Cd^+2^, and Cr^+6^ heavy metals ions and CS_MA (**A**), and CS_MAA (**B**) at pH 9.

The pH value is a major factor in the adsorption of the metal ions on the surface of the various chitosan samples. At an acidic pH value below 5, the free amino and hydroxyl groups of the chitosan backbone become protonated (–OH_2_^+^, –NH_3_^+^), which causes the metal ion adsorption to decrease. As the pH value increases, the protonation degree of the functional groups decreases, and the formation of complex coordination bonds between the functional groups and the metal ions takes place [44]. It should be stated that there are many modes for coordination of the polymer with the metal ions. The best pH value for the adsorption was determined to be 9 for all the chitosan samples, as shown in Figure 11. In general, whether grafting chitosan with methyl methacrylate or functionalizing it with maleic acid, they both had a significant impact on the removal of the metal ions as presented in Figure 11, with CS-MMA showing the highest percent removal. To better illustrate the adsorption process, the possible mechanisms for the metal ion adsorption onto the surface of CS-MMA are depicted in Figure 12. The adsorption of positively charged metal ions Ni^+2^, Pd^+2^, Cd^+2^, and Cr^+6^ on CS _MA and CS_MAA at pH 9 is expected to be dominated by two possible interactions.

One possible mechanism of these interactions is the chelation between the nitrogen atoms of the free chitosan amino groups and the metal ions. The other is the electrostatic attraction between either the negatively charged carboxylate ions of the chitosan side chains, in the case of CS_MA, or the negatively charged grafted acrylamide ions of the chitosan side chains, in the case of CS_MMA with the positively charged metal ions.

### 3.6. Regeneration

**Figure 13:**
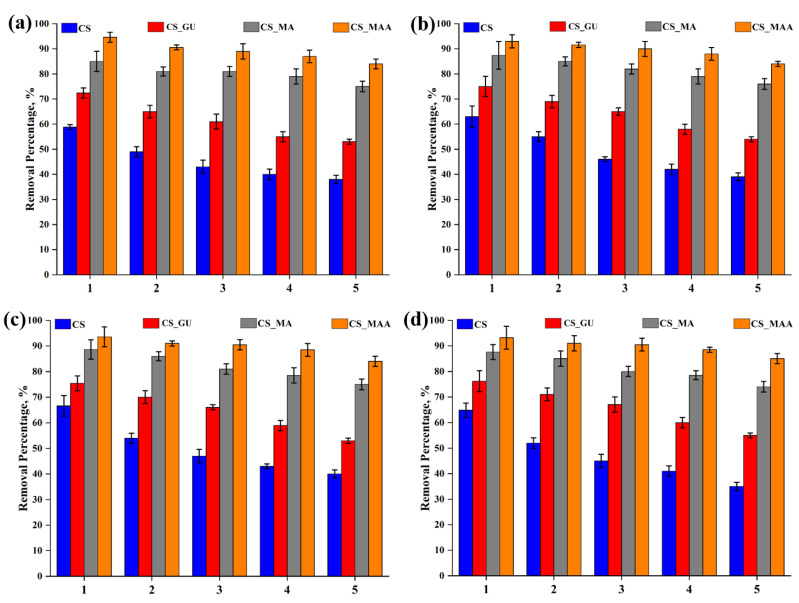
Cycles of the adsorption–desorption of nickel (**a**), lead (**b**), chromium (**c**), and cadmium (**d**) on the surface of poly(itaconic acid)-grafted chitosan beads.

Regeneration of the various chitosan adsorbents confirmed their reuse for five consecutive cycles, with an average percent removal dropping by only around 21% in the fifth cycle (Figure 13). The adsorption efficiency of CS and CS_GU, however, declined significantly more than CS_MA and CS_MMA did, which indicates the positive impact both the functionalization and the grafting copolymerization have on the regeneration of these bio-based adsorbents.

## 4. Conclusions

Adsorption experiments of four metal ions by four different chitosan-based polymeric adsorbents were conducted under different conditions. It was found that CS_MMA attained the maximum removal and uptake efficiency among all other polymeric adsorbents, reaching removal percentages of over 92% for all metal ions under consideration. Furthermore, the grafting copolymerization had a major positive effect on the crosslinked chitosan, increasing the removal percentage of the crosslinked chitosan samples from about 49% to 94%. The equilibrium isotherms for the metal ion adsorption fitted with the Langmuir isotherm. The uptake profiles for the adsorption were all well-fitted to a pseudo-first-order kinetic model. At lower pH values, the chitosan hydroxyl and amino groups are protonated, and the chitosan becomes a soluble cationic polymer that has a high charge density, which limits its ability to adsorb and remove the metal ions. Regeneration of the various chitosan adsorbents confirmed their reuse for five consecutive cycles, with an average percent removal dropping by only around 21% in the fifth cycle.

## Figures and Tables

**Table 1:** The thermodynamic parameters for the adsorption of nickel, lead, chromium and cadmium ions on the surface of the different chitosan-based polymeric adsorbents.

CS System	Thermodynamic Parameters for Nickel Ion Adsorption					Thermodynamic Parameters for Lead Ion Adsorption				
	ΔH (J/mol)	ΔS (J/mol·K)	ΔG (kJ/mol)	K_eq_	R^2^	ΔH (J/mol)	ΔS (J/mol·K)	ΔG (kJ/mol)	K_eq_	R^2^
CS_MA	663.63	37.60	−11.86	72.45	0.9637	609.57	37.35	−11.83	71.66	0.9494
CS_MAA	359.37	32.09	−10.33	41.66	0.9019	593.50	32.45	−10.21	39.97	0.905
**CS** **System**	**Thermodynamic parameters for chromium ion adsorption**					**Thermodynamic parameters for cadmium ion adsorption**				
	**Δ** **H** **(J/mol)**	**ΔS** **(J/mol·K)**	**ΔG** **(kJ/mol)**	**K_eq_**	**R^2^**	**ΔH** **(J/mol)**	**ΔS** **(J/mol·K)**	**ΔG** **(kJ/mol)**	**K_eq_**	**R^2^**
CS_MA	638.89	37.40	−11.81	71.32	0.9501	686.69	37.43	−11.78	70.39	0.9547
CS_MAA	1324.59	34.67	−10.22	40.12	0.9265	1225.40	34.03	−10.11	38.51	0.9209

**Table 2:** Kinetic parameters for the adsorption of the metal ions onto the surface of the best chitosan-based polymeric adsorbents CS_MA and CS_MMA using different kinetic models.

Kinetic Model	Kinetic Parameters	Nickel Ions		Lead Ions		Chromium Ions		Cadmium Ions	
		CS_MA	CS_MAA	CS_MA	CS_MAA	CS_MA	CS_MAA	CS_MA	CS_MAA
Pseudo-first-order	*k*_1_ (min^−1^)	0.0074	0.0062	−0.0078	−0.0062	−0.0064	−0.0062	−0.0064	−0.0062
	*q*_*e* (calc)_ (mg/g)	3.987	4.102	4.096	4.095	3.507	4.069	3.519	4.059
	*R* ^2^	0.9777	0.9923	0.9747	0.9926	0.9592	0.9917	0.9568	0.9936
Pseudo-second-order	*k*_2_ (kg/mg·min^−1^)	13.450	10.934	12.100	10.048	11.860	9.469	10.762	8.869
	*q*_*e* (calc)_ (mg/g)	0.016	0.019	0.017	0.020	0.017	0.021	0.018	0.021
	*R* ^2^	0.9323	0.955	0.9323	0.955	0.9469	0.9672	0.9549	0.9717
Intra-particle diffusion	*K_id_* (mg/(g·min^0.5^)	0.114	0.0858	0.1157	0.0946	0.1153	0.0937	0.1168	0.0960
	*R* ^2^	0.8862	0.8964	0.8898	0.9242	0.8884	0.9051	0.8938	0.9322

## Data Availability

Data are available within the manuscript and Supplementary Materials.

## Supplementary Materials

The following supporting information can be downloaded at: https://www.mdpi.com/article/10.3390/polym16121718/s1, Figure S1. Van’t Hoff plots for nickel (a), lead (b), chromium (c), and cadmium (d); Figure S2. The equilibrium isotherms for the adsorption of nickel (e), lead (f), chromium (g), and cadmium (h); Figure S3. Linear plots of the kinetic profiles for the uptake of nickel (a), lead (b), chromium (c), and cadmium (d) estimated using pseudo-first-order; Figure S4. Linear plots of the kinetic profiles for the uptake of nickel (a), lead (b), chromium (c), and cadmium (d) estimated using the pseudo-second-order; Figure S5. Linear plots of the kinetic profiles for the uptake of nickel (a), lead (b), chromium (c), and cadmium (d) estimated using the intra-particle diffusion model; Table S1: The thermodynamic parameters for the adsorption of nickel, lead, chromium and cadmium ions on the surface of the different chitosan-based polymeric adsorbents; Table S2: Kinetic parameters for the adsorption of the metal ions onto the surface of the different chitosan-based polymeric adsorbents using different kinetic models.
