# Supplementary material for: Novel High-Performance Functionalized and Grafted Bio-Based Chitosan Adsorbents for the Efficient and Selective Removal of Toxic Heavy Metals from Contaminated Water
Source: Polymers (Basel). 2024 Jun 16;16(12):1718. doi: 10.3390/polym16121718 (PMC11207307; doi:10.3390/polym16121718)
Supplement: Supplementary file 1 [file polymers-16-01718-s001.zip › polymers-3039500-supplementary.pdf]

Novel High-Performance Functionalized and Grafted Bio-Based Chitosan Adsorbents for the Efficient and Selective Removal of Toxic Heavy Metals from Contaminated Water

Mohammad Monir [1], Rasha E. Elsayed [1], Rasha A. Azzam [2] and Tarek M. Madkour [1,*]

[1] Department of Chemistry, School of Sciences and Engineering, The American University in Cairo, AUC Avenue, New Cairo, Cairo 11835, Egypt; mohammadmonir@aucegypt.edu (M.M.); r.essam@aucegypt.edu (R.E.E.)

[2] Department of Chemistry, Faculty of Science, Helwan University, Cairo 11795, Egypt; rasha_azzam@science.helwan.edu.eg

* Correspondence: tarekmadkour@aucegypt.edu

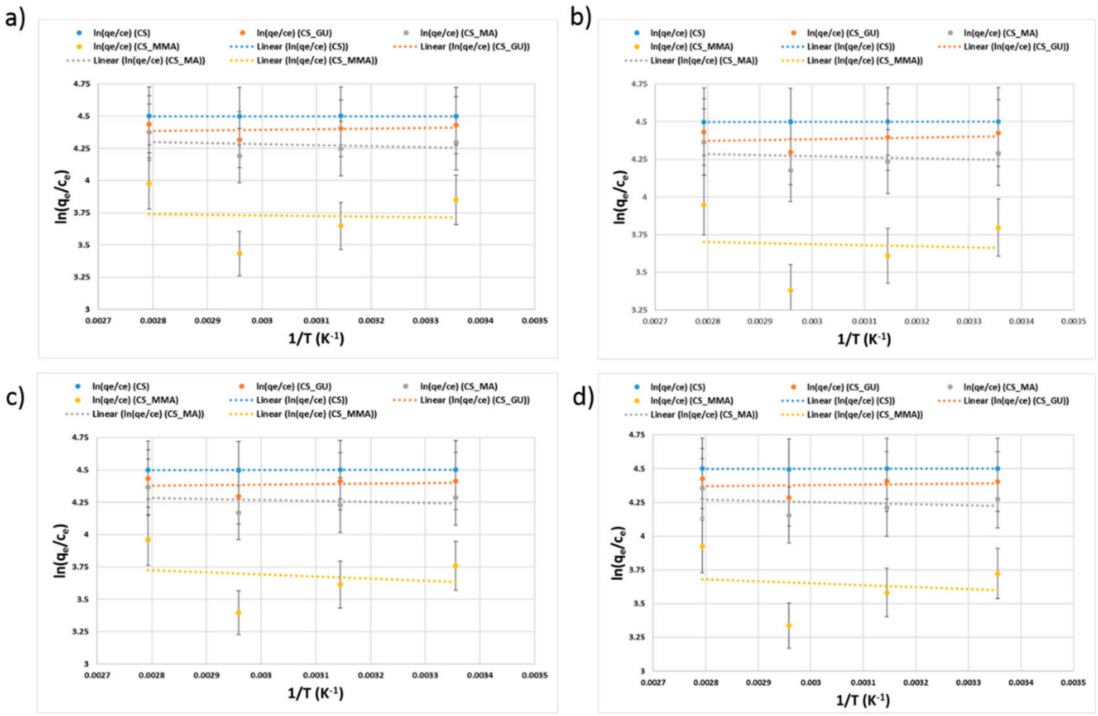

Figure S1. Van't Hoff plots for nickel (a), lead (b), chromium (c), and cadmium (d).

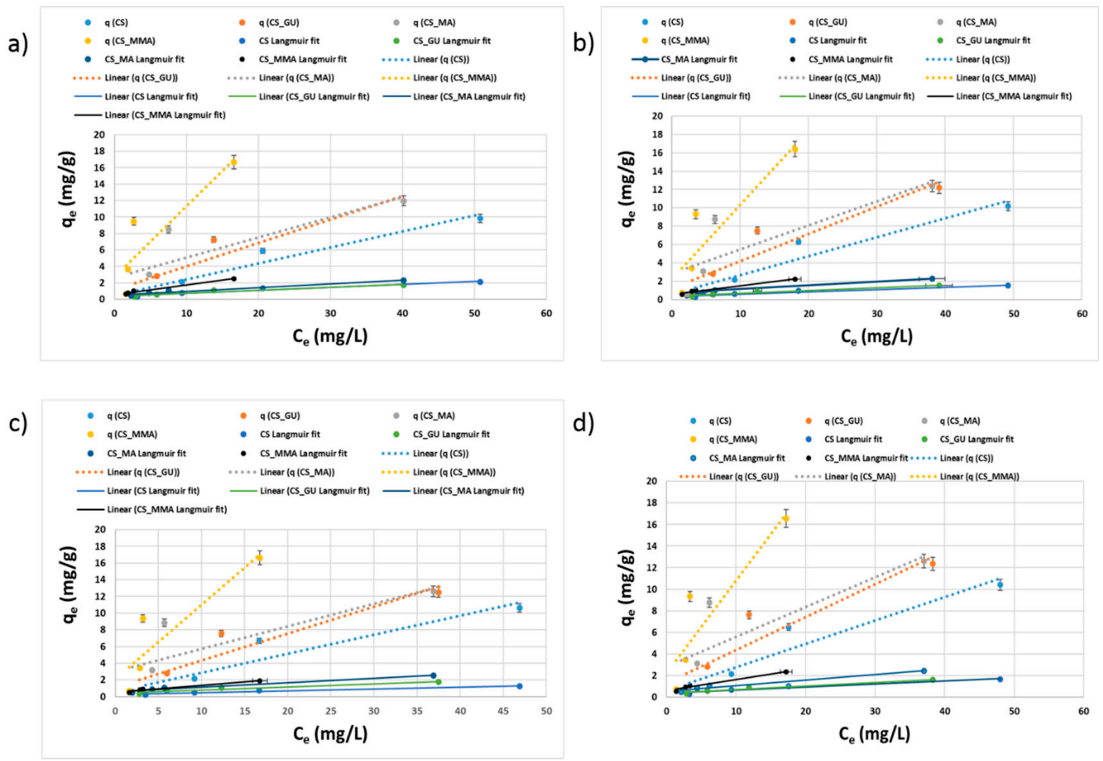

Figure S2. The equilibrium isotherms for the adsorption of nickel (e), lead (f), chromium (g), and cadmium (h).

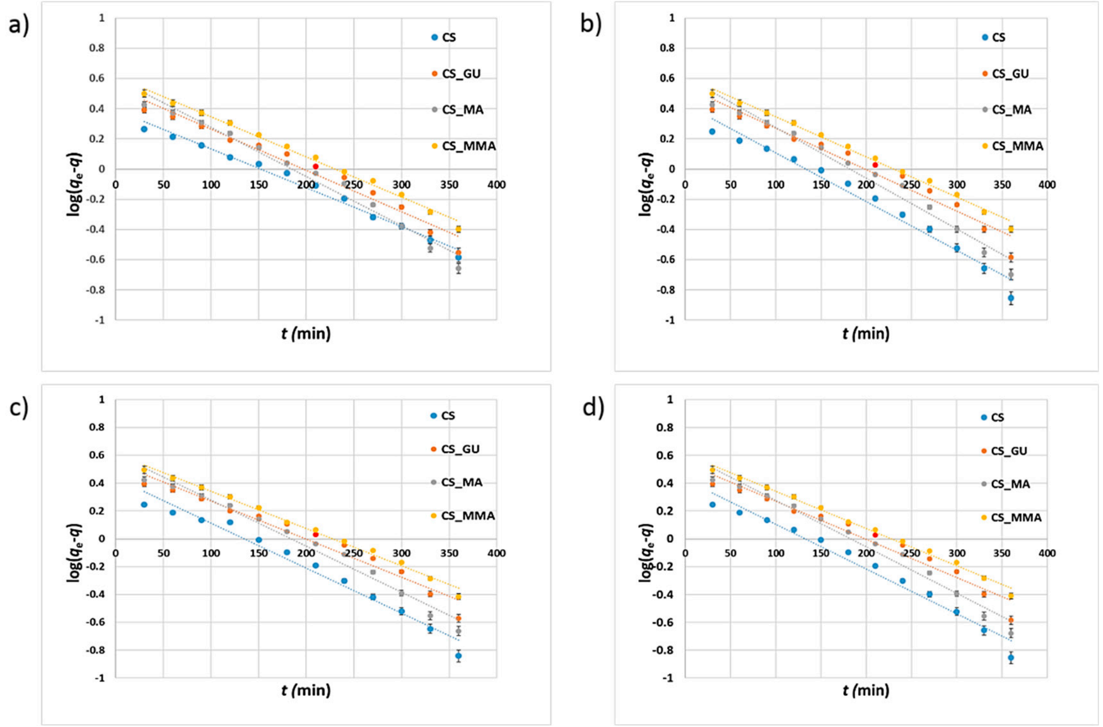

Figure S3. Linear plots of the kinetic profiles for the uptake of nickel (a), lead (b), chromium (c), and cadmium (d) estimated using pseudo-first-order.

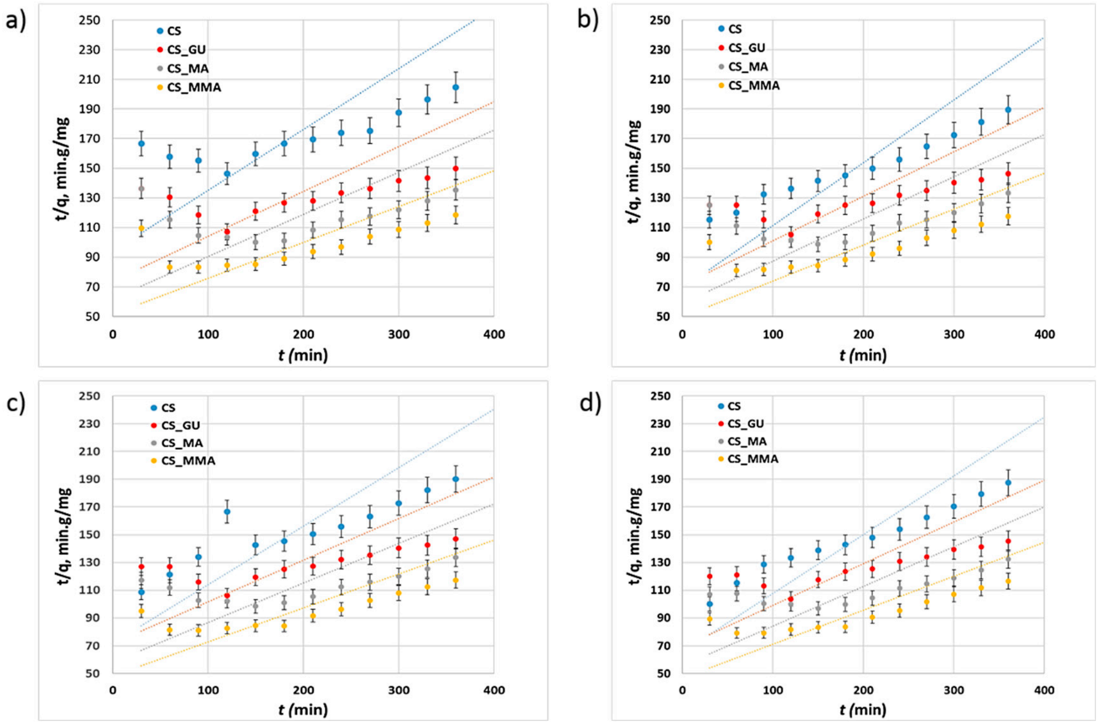

Figure S4. Linear plots of the kinetic profiles for the uptake of nickel (a), lead (b), chromium (c), and cadmium (d) estimated using the pseudo-second-order.

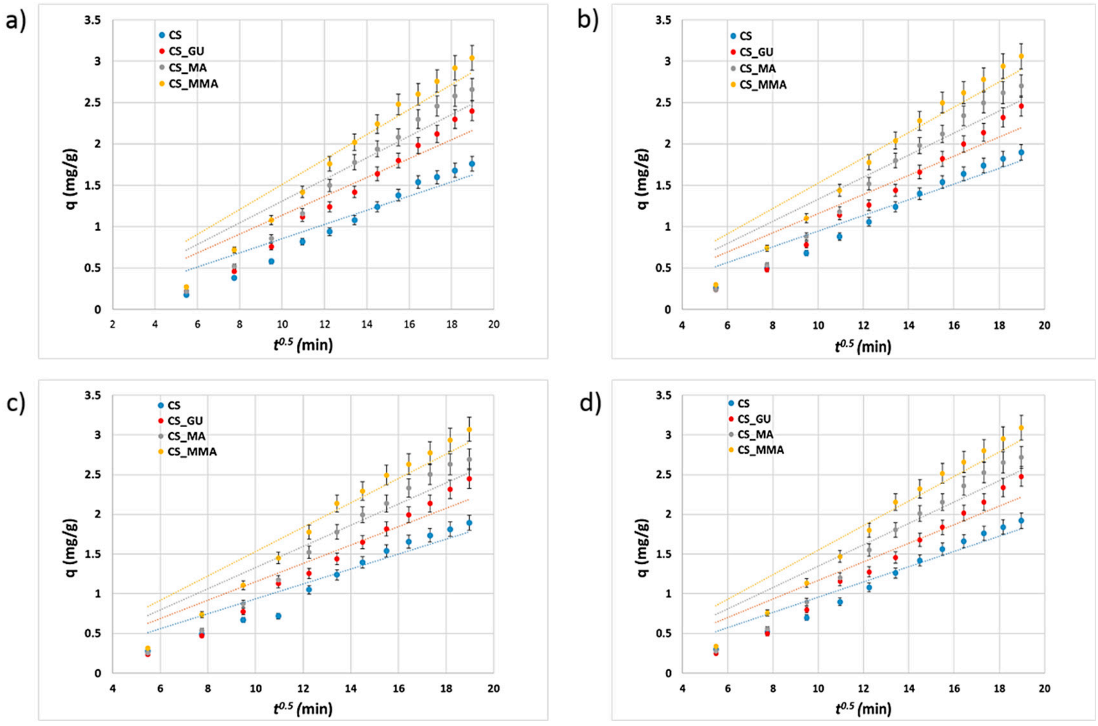

Figure S5. Linear plots of the kinetic profiles for the uptake of nickel (a), lead (b), chromium (c), and cadmium (d) estimated using the intra-particle diffusion model.

Table S1: The thermodynamic parameters for the adsorption of nickel, lead, chromium and cadmium ions on the surface of the different chitosan-based polymeric adsorbents.

CS System	Thermodynamic parameters for nickel ions adsorption					Thermodynamic parameters for lead ions adsorption				
	ΔH (J/mol)	ΔS (J/mol.K)	ΔG* (kJ/mol)	K_{eq}	R^2	ΔH (J/mol)	ΔS (J/mol.K)	ΔG* (kJ/mol)	K_{eq}	R^2
**CS**	-13.34	37.45	-12.46	90.03	0.9423	-46.72	37.27	-12.46	90.01	0.9656
**CS_GU**	-365.66	35.44	-12.17	81.01	0.9381	-450.61	35.10	-12.14	80.17	0.9444
**CS System**	**Thermodynamic parameters for chromium ions adsorption**					**Thermodynamic parameters for cadmium ions adsorption**				
	ΔH (J/mol)	ΔS (J/mol.K)	ΔG* (kJ/mol)	K_{eq}	R^2	ΔH (J/mol)	ΔS (J/mol.K)	ΔG* (kJ/mol)	K_{eq}	R^2
**CS**	-41.60	37.29	-12.46	90.01	0.9121	-47.14	37.27	-12.46	89.98	0.9013
**CS_GU**	-324.73	35.50	-12.15	80.43	0.9232	-309.06	35.48	-12.12	79.72	0.9547

Table S2: Kinetic parameters for the adsorption of the metal ions onto the surface of the different chitosan-based polymeric adsorbents using different kinetic models.

**Kinetic model**	**Kinetic parameters**	**Nickel ions**		**Lead ions**		**Chromium ions**		**Cadmium ions**	
		**CS**	**CS_GU**	**CS**	**CS_GU**	CS	**CS_GU**	**CS**	**CS_GU**
**Pseudo-first order**	k_1 (min^{-1})	0.0060	0.0062	-0.0074	-0.0064	-0.0074	-0.0076	-0.0074	-0.0076
	$q_{e\,(calc)}$ (mg/g)	2.451	3.455	2.681	3.518	2.728	4.061	2.671	4.078
	R^2	**0.9868**	**0.9665**	**0.973**	**0.9567**	0.9721	**0.9772**	0.9723	**0.976**
**Pseudo-second order**	k_2 (kg/mg.min^{-1})	21.499	17.868	11.216	16.701	12.247	17.145	10.041	15.805
	$q_{e\,(calc)}$ (mg/g)	0.011	0.014	0.014	0.014	0.014	0.014	0.015	0.015
	R^2	0.9628	0.9485	0.9628	0.9485	**0.9741**	0.9545	**0.983**	0.9605
**Intra-particle diffusion**	K_{id} (mg/(g.min$^{0.5}$)	0.1511	0.131	0.1526	0.1332	0.1534	0.1332	0.1549	0.1348
	R^2	0.9092	0.8849	0.9136	0.8877	0.9149	0.8878	0.9191	0.8934
